# Seasonal and climatic variation in the incidence of adult acute appendicitis: a seven year longitudinal analysis

**DOI:** 10.1186/s12873-020-00321-2

**Published:** 2020-04-07

**Authors:** Thomas James York

**Affiliations:** 1 St Mary’s Hospital, Imperial College Healthcare NHS Trust, Praed Street, London, W2 1NY UK; 2 Harrogate and District Hospital, Harrogate and District NHS Foundation Trust, Park Road, Harrogate, Lancaster, HG2 7SX UK

## Abstract

**Background:**

Acute appendicitis represents an extremely common surgical emergency, yet its aetiology remains uncertain. A multifactorial understanding of its causation has emerged along with increasing evidence of seasonal variation.

This study seeks to find evidence for such a circannual trend within the United Kingdom (UK), and further assess key meteorological indicators which may be causative of any such variation.

**Methods:**

The patient records of a region health body in the North East of England were retrospectively assessed over a 7-year period. The incident cases of acute appendicitis were recorded and averaged by month before undergoing statistical analysis for variation and correlation with average temperature, sunlight hours, and rainfall.

**Results:**

The incidence of acute appendicitis revealed significant seasonal variation with only 38 incident cases in the months of January compared to 73 in July, a 92.1% increase.

Only a weak correlation was seen between incidence and average sunlight hours/rainfall, however a significant, positive correlation was found between incidence and average temperature (*r = 0.58, p = 0.048*).

**Conclusion:**

Compelling evidence is found to support the existence of a circannual trend for acute appendicitis. Data suggests a seasonal peak in the month of July, accompanied by a low in January, a finding that develops the understanding of this trend from previously equivocal research in the UK.

A clear correlation is also established between the incidence of acute appendicitis and average temperature. The 92.1% increase between the coolest and warmest months suggests a greater magnitude for this as a risk factor than has previously been shown.

## Background

Acute inflammation of the vermiform appendix represents one of the most common surgical emergencies [1]. The lifetime risk of developing acute appendicitis is significant, 8.6% in males and 6.7% in females [2], yet the aetiology of the condition remains uncertain. Factors including a low fibre diet [3], male gender, and age between late teens and early fifties [4] have all been shown to increase risk.

Alongside these influences, there is a growing evidence base to suggest that the incidence of acute appendicitis displays seasonal variation [1, 5, 6]. With more than a quarter of a million appendicectomies performed annually in the United States (US) alone [7], an understanding of these seasonal and climatic factors has the potential to improve current standards of risk assessment and workload planning.

This study seeks to address two primary aims. The first of these was to establish whether or not significant seasonal variation in acute appendicitis can be demonstrated in the United Kingdom; a country with notably variable weather conditions [8] and limited existing research. Secondly, the author aims to use meteorological data to identify specific climatic features which may be causative of any such variation.

## Methods

The electronic patient records of Harrogate and District NHS Foundation Trust (HDFT) were retrospectively scrutinised for the 7-year period from 01/09/2012 to 31/08/2019. HDFT serves a predominantly white British population, across mixed rural and urban centres in the North East of England. Office for National Statistics (ONS) records for the Borough of Harrogate show a population of 158,237 in 2012. In 2018, the latest available data, this had remained largely stable with a population of 160,533. Population density for the district is representative of its chiefly rural population at 120 people per square kilometre.

Inclusion criteria were set to identify all admission episodes for patients over the age of the 18 and with an associated diagnosis of acute appendicitis. This revealed a total of 703 entries which were further analysed to create an anonymised database featuring: date of admission, duration of admission, gender, and age.

Following this, a date-resolved analysis of the distribution of entries was performed. The total admissions for acute appendicitis were collated by calendar month in order to examine the database for seasonal variation. A further analysis was performed using Met Office records to establish an average rainfall, number of sunshine hours, and temperature for each included month. These were taken from the nearby Whitby (80 km) and Bradford (26 km) weather stations; the two most proximal sites to the HDFT catchment area and sharing its climatic features, bordering its margins to the east and west.

An expected number of cumulative admissions per month (2012–2019) was established on the basis of a null hypothesis. This was taken to be that the incidence of acute appendicitis is normally distributed across the year; that there is no seasonal variation over the 12 months and an equal number of episodes should be recorded in each month regardless of the analysed independent variables. An expected number of incident cases for each month was therefore one twelfth of the total figure, 703/12 = 58.6 episodes per calendar month.

Biostatistical analysis was then performed to assess the significance of observed variation from the expected value. Chi Square *P-value* testing was employed with the threshold for significance being set at the conventional value of *P = 0.05*. The observed monthly incident cases of acute appendicitis were also compared against the three recorded meteorological indices using Pearson correlation coefficient, this allowed the association between climatic features and incidence to be assessed.

This research was reviewed and approved by the local audit committee and, as a purely retrospective analysis, did not involve patient intervention or contact.

## Results

Of the 703 included episodes of acute appendicitis, 366 (52.1%) were associated with male patients and the remaining 337 (47.9%) with female patients. The average admission duration across the study was 3.4 days with an average patient age of 38.

In accordance with National Institute for Health and Care Excellence (NICE) guidelines, all 703 patients underwent either a laparoscopic or open appendicectomy.

### Date-resolved analysis

January was found to have the lowest observed incident cases of acute appendicitis with only 38 (5.4%) of the episodes occurring in that month, this was a 35.1% reduction on the average. Biostatistical analysis suggested it to be a significantly reduced proportion (*p = 0.005)*.

The month with the highest observed incidence of acute appendicitis was July, recording 73 episodes (10.4%) over the study period. When compared to the expected value this represented a significant increase of 24.6% (*p = 0.049)*.

**Fig. 1:**
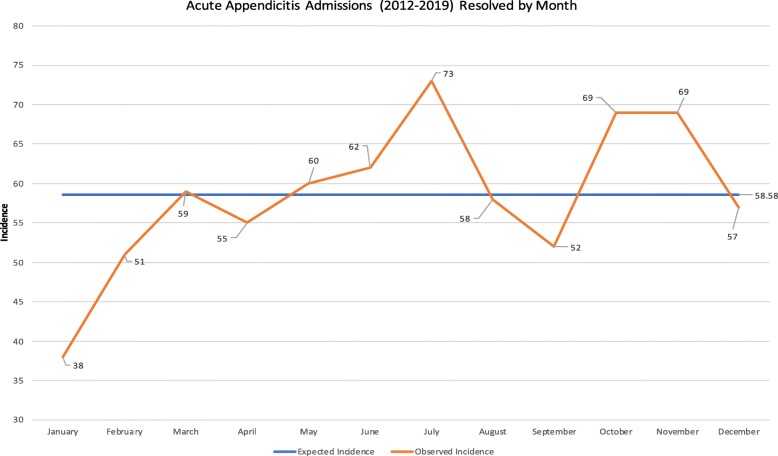
Displays cumulative incidence of acute appendicitis admissions from 2012 to 2019 (resolved by month) against the expected, mean incidence

An annual trend was observed with a lower than expected incidence of acute appendicitis during the winter months (Dec, Jan, Feb), compared to an increased incidence in the late spring and summer (May, Jun, Jul) see Fig. 1.

### Sunlight hours

**Fig. 2:**
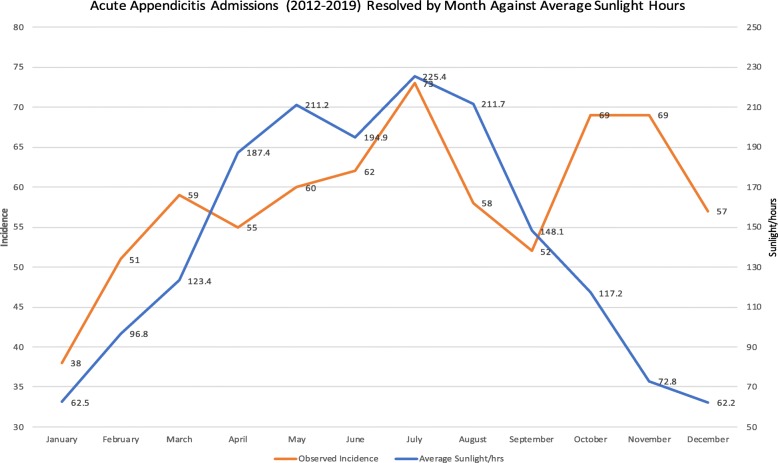
Displays cumulative incidence of acute appendicitis admissions from 2012 to 2019 against the average number of monthly sunlight hours for the same period and geographic area

As could be expected, the average sunlight hours were found to be highest during the summer months and lowest during the winter months see Fig. 2. This climatic indicator displayed high annual variation, the maxima (62.5 h) representing 360.1% of the minima (225.4 h).

In January and December there were an average of 62.5 h and 62.2 h of sunlight respectively, the lowest two values. This was found to correlate with reduced rates of acute appendicitis compared to the expected value, 48 in December and 38 in January.

The highest number of incident cases, 73 episodes (10.4%) in July, was found to coincide with the greatest average monthly sunlight hours (225.4 h).

When Pearson correlation coefficient testing was performed, average sunlight hours and incidence of acute appendicitis were found to be weakly positively correlated (*r = 0.37, p = 0.24)*.

### Rainfall

The maxima of average rainfall, 73.0 mm in January, was found to coincide with the lowest incidence of acute appendicitis.

**Fig. 3:**
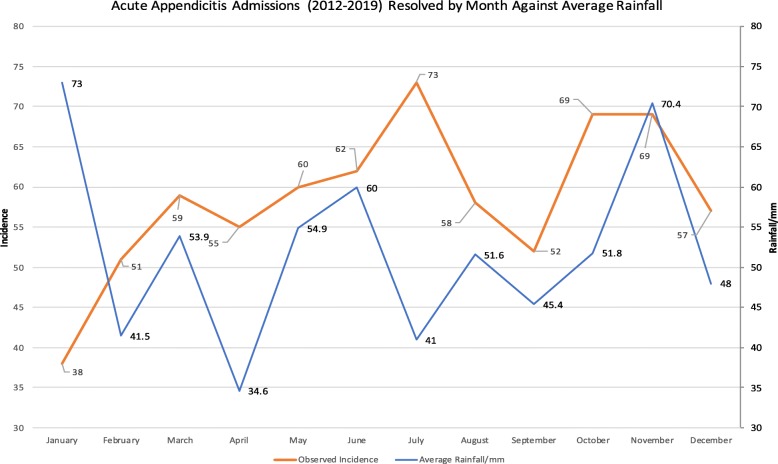
Displays cumulative incidence of acute appendicitis admissions from 2012 to 2019 against the average monthly rainfall for the same period and geographic area

However, the lowest average rainfall was seen in April (34.6 mm) which was associated with 55 episodes. This fell within statistically insignificant deviation from expected values (*p = 0.62*). The second lowest average rainfall was seen in July (41.0 mm), the month with the highest incidence of acute appendicitis see Fig. 3.

Annual variation in this climatic indicator was lower than sunlight hours, with the maxima 210.9% of the minima.

Statistical testing revealed a weakly negative correlation between average rainfall and incidence of appendicitis (*r = − 0.15, p = 0.64*).

### Temperature

Average monthly temperature was calculated as the mean of the average maximum and minimum temperatures, in accordance with Met Office guidance [9].

**Fig. 4:**
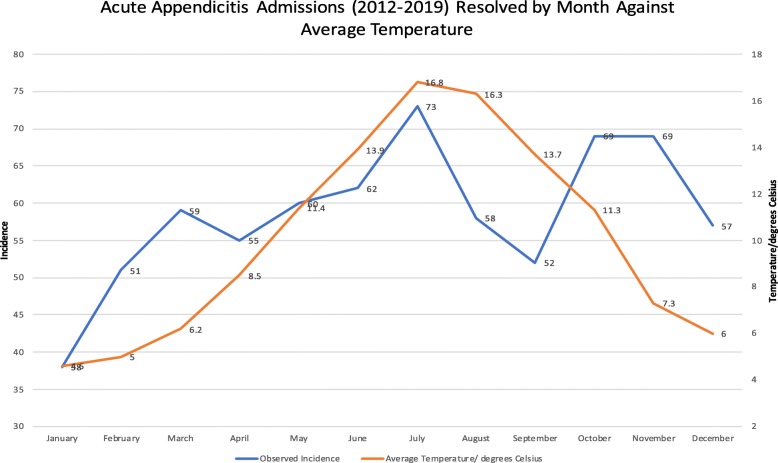
Displays cumulative incidence of acute appendicitis admissions from 2012 to 2019 against the average monthly temperature for the same period and geographic area

The lowest average temperature was noted in January (4.6 C), coinciding with the lowest incidence of acute appendicitis (38). Likewise, the highest average temperate was seen in July (16.8 C), the month with the highest observed incidence see Fig. 4.

Temperature showed very similar annual variation to sunlight hours, with the maxima 365.2% of the minima.

Of the studied climatic indicators, average temperature showed the greatest association with the incidence of acute appendicitis. A moderately positive correlation was found between the two, meeting the threshold for statistical significance (*r = 0.58, p = 0.048*). An increase in temperature of 12.2 degrees (from lowest to highest) was associated with a 92.1% increase in the monthly incidence.

## Discussion

The demographic information from the contributing 703 admission episodes appears consistent with that observed in prior work. The slight male preponderance (52.1% male, 47.9% female) has been similarly noted in comparable international studies [5, 10] and suggests the sample is more broadly representative of patients with acute appendicitis. This is further supported by an average patient age of 38 years, consistent with the observations of national health databases in the UK and US [4, 7].

Numerous studies have shown seasonal variation in acute appendicitis [1, 2, 5, 6, 10] and a recent meta-analysis, representing some of the strongest evidence for this, showed a peak incidence in the summer months for 9 of the 11 included studies [11]. However, research from the UK has been sparse in this field and past work has not shown clear circannual variation [12]. This study contrasts that and is able to demonstrate significant evidence that a seasonal trend, with a maximum incidence in July and minimum incidence in January, is observable in the UK.

The cause for this seasonal variation remains controversial with evidence suggesting possible links to behavioural changes, in particular the increased consumption of low fibre foods [3, 13] and alcohol [14] as people spend more time socialising outdoors. It has been hypothesised that these factors increase the risk of gastrointestinal infection due to delayed bowel transit, thereby predisposing to acute appendicitis [11]. Whilst this may go some way to explaining seasonal variation, increased alcohol consumption does not appear to correspond with the summer peak in the UK. Here studies have shown alcohol intake to be at its highest in December and January [15], when incidence of appendicitis is relatively low; in this study the 5th lowest and lowest incidence months respectively. Equally, whilst international research has shown fibre consumption to increase in the summer months [16], diet is highly variable between countries and there is limited domestic evidence to demonstrate such a trend [17].

Given the results of this study, the author proposes that temperature itself may be the causative factor for seasonal variation in acute appendicitis. A significant, positive correlation was observed between rising temperatures and an increased incidence. Unlike seasonal behavioural changes, this may also account for the observation of a winter low in appendicitis; cooler weather acting as a relatively protective factor. Such a conclusion is supported by work conducted in similar northern European populations, where an increase of 10 degrees Celsius was linked to a 4% rise in acute appendicitis [6]. This study appears to demonstrate the possibility of a much more marked effect, with a 92.1% increase occurring over the 12.2 degrees Celsius range from lowest to highest average temperature.

Evidence to suggest a role for sunlight hours and rainfall in seasonal variation was found to be substantially weaker. Neither met the threshold for statistically significant correlation and it is hypothesised that any concurrent trend is most likely a function of their close relationship with average temperature.

### Limitations

The relative ethnic homogeneity of the sample group, a consequence of a predominantly white British patient demographic at HDFT, has an uncertain influence on the results of this study. Previous work has shown significant variation in appendicitis across different ethnicities, but no absolute preponderance has been established [1, 18]. Further research is required to control for this.

Although this study was able to assess admissions over a 7-year period it was not possible to extend beyond this due to limitations in the electronic patient records. Statistical analysis has suggested this was able to yield significant results however more longitudinal investigation would likely serve to increase the confidence with which temperature can be judged to influence acute appendicitis.

One further weakness of this study was the exclusion of patients under the age of 18. The decision not to include paediatric cases of acute appendicitis was predicated on the presence of seasonally concurrent variables such as school holidays and term dates. The effect of these would have had an unknown bearing on results without ready means to control for them. Further research is indicated to explore if the seasonal variation demonstrated in this work is also observable in paediatric populations.

## Conclusions

This study identifies clear evidence to support wider findings of seasonal variation in the incidence of acute appendicitis. In particular it suggests a seasonal peak in the month of July, accompanied by a low in January, a finding that develops the understanding of this trend from previously equivocal research in the UK.

A significant, positive correlation is also established between the incidence of acute appendicitis and average temperature. An increase of 92.1% in admissions between the coldest and warmest months not only indicates rising temperature to be a risk factor for the condition, but also suggests a potential magnitude for this effect beyond that which has previously been shown.

## Data Availability

All data used in the creation of this work will be made available upon request.
